# Efficacy of a Metal Artifact Reduction Algorithm in CBCT Images of Teeth With Ceramic Brackets With/Without Coated Archwires: An In Vitro Study

**DOI:** 10.1002/cre2.70112

**Published:** 2025-03-16

**Authors:** Parisa Soltani, Mariangela Cernera, Marzie Kachuie, Amirhossein Moaddabi, Mehran Khoramian, Gianrico Spagnuolo, Niccolò Giuseppe Armogida, Carlo Rengo

**Affiliations:** ^1^ Department of Oral and Maxillofacial Radiology Dental Implants Research Center, Dental Research Institute, School of Dentistry, Isfahan University of Medical Sciences Isfahan Iran; ^2^ Department of Neurosciences Reproductive and Odontostomatological Science, University of Naples “Federico II” Naples Italy; ^3^ Department of Orthodontics Dental Research Center, School of Dentistry, Isfahan University of Medical Sciences Isfahan Iran; ^4^ Department of Oral and Maxillofacial Surgery Dental Research Center, Mazandaran University of Medical Sciences Sari Iran; ^5^ Student Research Committee School of Dentistry, Isfahan University of Medical Sciences Isfahan Iran

**Keywords:** artifacts, cone‐beam computed tomography, orthodontic brackets

## Abstract

**Objective:**

This study aimed to assess the efficacy of a metal artifact reduction (MAR) algorithm for cone‐beam computed tomography (CBCT) scans of teeth with ceramic brackets with/without coated archwires.

**Material and Methods:**

In this in vitro study, 10 ceramic brackets were bonded to maxillary anterior teeth from the second premolar of one side to the second premolar of the other side on a dry human skull. CBCT scans (85 kVp, 8 mA, 14.5 s) were taken twice in the presence and absence of coated nickel–titanium (NiTi) archwires. The skull was placed in a water container for soft tissue simulation during scanning. The images were reconstructed with and without the MAR algorithm, and imported to ImageJ software in DICOM format to calculate the contrast‐to‐noise ratio (CNR) at 15‐, 20‐, and 25‐mm distances from the tooth center. Data were analyzed by independent *t*‐test, ANOVA, and Bonferroni test (α = 0.05).

**Results:**

Application of the MAR algorithm had no significant effect on the CNR in the presence or absence of archwire (*p* > 0.05). Significant differences were found in the CNR according to tooth type and distance from the tooth center, such that the CNR significantly increased in farther distances (*p* < 0.05).

**Conclusions:**

Within the limitations of this in vitro study, the results showed that the application of the MAR algorithm had no significant efficacy in improving the quality of CBCT scans of teeth with ceramic brackets with/without coated archwire.

## 1 Introduction

Fixed orthodontic treatment is commonly performed for correction of malocclusion and improvement of oral health, function, and esthetics (Ren et al. 2014). Brackets, bands, ligature materials, and archwires are the main components of fixed orthodontic appliances (Kumar et al. 2013). Orthodontic brackets are fabricated from different materials such as stainless steel and titanium metals, and nonmetal materials such as plastic, ceramic, and plastic composite resins (Mundhada et al. 2023). Orthodontic brackets can be bonded to the labial or lingual surface of the teeth (Rahiotis and Schricker 2017). Archwires are usually fabricated from nickel–titanium (NiTi) or stainless steel (Mundhada et al. 2023).

Radiographic modalities are a key parameter in dental diagnosis and treatment (Mundhada et al. 2023). Panoramic radiography and lateral cephalometry used to be the standard imaging modalities in orthodontics. However, they have shortcomings due to their two‐dimensional nature, such as superimposition of structures, incorrect position identification, and increasing the patient radiation dose by necessitating multiple images (Ting et al. 2020).

Cone‐beam computed tomography (CBCT) provides high‐quality three‐dimensional images of the hard tissues with lower radiation dose compared to computed tomography (CT); thus, it has gained growing popularity in orthodontics (Pauwels et al. 2013). CBCT is increasingly used as a replacement for the conventional computed tomography and two‐dimensional imaging modalities in dentistry (Sheikhi et al. 2021). CBCT has several applications in orthodontics. It can be used for evaluation of impacted or ectopic teeth, airways, and mini‐implant sites, analysis of craniofacial deformities, preoperative planning for orthognathic surgery, and detection of root resorption, among others (Abdelkarim 2019). CBCT is known as an efficient imaging modality in many dental fields and particularly orthodontics since it provides high‐resolution three‐dimensional images of the hard tissues with a relatively low radiation dose (Soltani, Moaddabi, et al. 2024). Nonetheless, artifacts are known as a major drawback of CBCT (Soltani, Moaddabi, et al. 2024; Soltani, Devlin, et al. 2024). CBCT artifacts can be categorized into four main groups of inherent artifacts, procedure‐related artifacts, introduced artifacts, and motion artifacts. Metal artifacts are generated due to different absorption of low‐energy photons by metal objects, and are grouped under the category of introduced artifacts (Lam and Mallya 2025). They can decrease the quality of the image, and distort the image of structures and defects around metal objects (Khosravifard et al. 2021; Lucca et al. 2024). Beam hardening is a phenomenon related to metal artifacts, which occurs due to different absorption of low‐energy and high‐energy photons by metal objects. This phenomenon can generate two types of artifacts: (i) cupping or blooming artifacts, which occur due to differential absorption of photons by metal structures, and distort the image in areas close to metal objects. This type of artifact often manifests by increasing the dimensions and distortion of the shape of metal objects. (ii) Extinction or missing value artifacts, which cause dark lines and streaks between two metal objects due to a difference in photon absorption and beam hardening around metal objects (Shavakhi et al. 2024). These artifacts can influence the image quality and diagnostic potential of CBCT images for different diagnostic tasks (Soltani, Moaddabi, et al. 2024; Soltani, Devlin, et al. 2024b; Abdinian et al. 2020; Hilgert et al. 2025). Therefore, the elimination of metal artifacts in CBCT images has become an increasingly active and significant area of research aiming to enhance image quality and diagnostic accuracy by developing advanced algorithms and techniques to mitigate the distortions and inaccuracies caused by metal implants and objects. The metal artifact reduction (MAR) algorithms were developed to decrease the effect of artifacts and improve image quality. These algorithms are extensively used for diagnostic purposes when metal objects are present in the field of view (FOV)+ and are applied in different steps of image reconstruction (Candemil et al. 2019). Application of these algorithms may significantly decrease or eliminate artifacts, and enhance the diagnostic accuracy (Queiroz et al. 2018; Korpics et al. 2016). Application of MAR algorithms is particularly important in the presence of metal objects such as orthodontic brackets in the CBCT FOV (Hirschinger et al. 2015). Nonetheless, a limited number of studies are available on artifact generation by different types of orthodontic brackets, and the efficacy of different MAR algorithms to decrease them. Search of the literature by the authors yielded no study on the efficacy of MAR algorithms for reduction of artifacts generated by ceramic brackets and coated archwires. Thus, this study aimed to assess the efficacy of a MAR algorithm for CBCT scans of teeth with ceramic brackets with/without coated NiTi archwires. The null hypothesis of the study was that the tested MAR algorithm would have no significant efficacy for reduction of artifacts on CBCT scans of teeth with ceramic brackets with/without coated NiTi archwires.

## 2 Materials and Methods

This in vitro experimental study was conducted on 10 ceramic brackets bonded to maxillary anterior teeth from the second premolar of one side to the second premolar of the other side in a dry human skull. The study protocol was approved by the ethics committee of the Isfahan University of Medical Sciences (Number: IR.MUI.DHMT. REC.1403.044, Approval date: 10/6/2024).

### 2.1 Sample Size

The sample size was calculated using the following formula:

$$n=\frac{{\left(Z_{1-\frac{\alpha }{2}}+Z_{1-\beta }\right)}^{2}(\sigma_{1}^{2}+\sigma_{2}^{2})}{d^{2}}$$

Assuming alpha = 0.05, beta = 0.2, and study power of 0.80, two repetitions of CBCT scans were required from 10 teeth to find a minimum significant difference of 0.8 between the mean values.

### 2.2 Specimen Preparation

A dry human skull with sound, caries‐free maxillary anterior teeth was used in this study. Ten ceramic brackets (American Orthodontics, Sheboygan, WI, USA) were carefully bonded to the buccal surface of the teeth from the second premolar of one side to the other. For this purpose, the enamel surface was etched with 37% phosphoric acid for 30 s, rinsed with water, and air‐dried. The bonding agent (Brace Paste; American Orthodontics, Sheboygan, WI, USA) was applied on the enamel surface for 30 s and air‐thinned. Brackets were positioned on the enamel surface, and light‐curing was performed for 30 s using a curing unit (Valo Cordless, Ultradent, South Jordan, UT, USA) with a light intensity of 480 mW/cm^2^.

To simulate fixed orthodontic treatment, coated NiTi archwires (American Orthodontics, Sheboygan, WI, USA) measuring 0.022 × 0.016 inch were engaged in the brackets using clear O‐rings (American Orthodontics, Sheboygan, WI, USA).

### 2.3 CBCT Acquisition Parameters

The skull was placed in a water container for soft tissue simulation during scanning. CBCT scans (Papaya 3D, Genoray, Seongnam‐Si, Korea) were taken from the assembly in two modes—with and without activation of the MAR algorithm—using the following exposure settings: 85 kVp tube potential, 8 mA tube current, 14.5 s scanning time, 14 × 8 cm FOV, and 75 μm voxel size.

### 2.4 CBCT Scanning Circumstances

In each mode, CBCT scans were taken in the following circumstances:

– Teeth without a bracket and wire
– Teeth with buccal brackets
– Teeth with buccal brackets and archwire

Two CBCT scans were taken from each of the abovementioned circumstances. The two scans were saved once without the MAR algorithm (i.e., SMARF in this particular software system) and once with the activation of the MAR algorithm.

### 2.5 Image Processing

**Figure 1:**
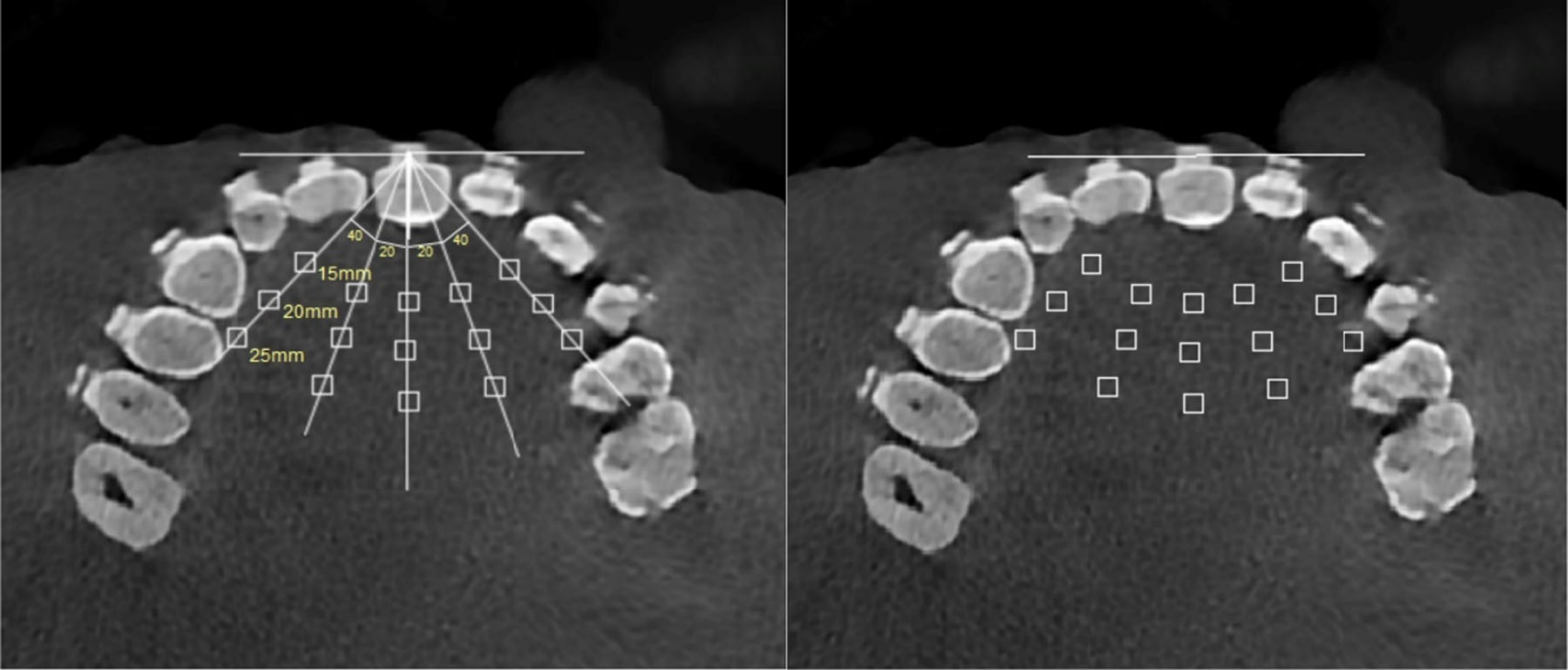
Analysis of axial images using ImageJ software.

The image analysis procedures used in this study were adopted from similar studies by Shavakhi et al. (2024) and Fontenele et al. (2020). The CBCT scans were imported from the native scanner software, i.e., Theia (Genoray, Seongnam‐Si, Korea), to OnDemand software (Cybermed, Seoul, Korea). The DICOM file was then imported to ImageJ software (NIH, Maryland, USA). One particular axial image visualizing the mid‐level of the bracket was selected as the reference for analysis. All measurements in different groups were made on this axial image. The measurements, including the mean and standard deviation of gray values at the region of interest (ROI) of different teeth, were later used to calculate the CNR. A trained senior dental student, under the supervision of an oral and maxillofacial radiologist, made all the measurements. For this purpose, a line was drawn from the bracket center along the buccal surface of the tooth. A vertical line was drawn perpendicular to this line, and 15 ROIs measuring 2 × 2 mm were considered for the brackets of central incisor to second premolar teeth of the right side at 15‐, 20‐, and 25‐mm distances from the tooth center with 20‐, 40‐, and 90‐degree angles at each side relative to the vertical line (Figure 1).

### 2.6 Calculation of CNR

To calculate the CNR, an ROI with the abovementioned dimensions was selected in the water surrounding the dry skull, and the mean and standard deviation of the gray value of this ROI were recorded as control. The CNR was calculated using the following equation:

$$\mathrm{CNR}=\frac{\left|{\mathrm{Mean}}_{\mathrm{bracket}}-{\mathrm{Mean}}_{\mathrm{control}}\right|}{\sqrt{{\mathrm{SD}}_{\mathrm{bracket}}^{2}+{\mathrm{SD}}_{\mathrm{control}}^{2}}}$$

The CNR values of all ROIs from various angles at each distance (15 mm, 20 mm, and 25 mm) were averaged to provide a single CNR value for each distance. This single CNR value was considered the primary outcome measure.

### 2.7 Statistical Analysis

The normality of data distribution and presence of outliers were analyzed using the Shapiro‐Wilk test, and skewness and kurtosis. Due to normal distribution of data and insignificant skewness, comparisons were made by independent t‐test, one‐way ANOVA, and Bonferroni test (for pairwise comparisons) using STATA software at 0.05 level of significance.

## 3 Results

Table 1 presents the mean CNR in the two groups with and without the MAR algorithm in different modes. As shown, in presence of ceramic brackets and coated NiTi archwire, the mean CNR is slightly higher when MAR is enabled (0.9789 vs. 0.9697), but the difference is not significant (*p* = 0.94). In other words, the activation of MAR has no significant effect on the artifacts of CBCT images of teeth with ceramic brackets and coated archwire.

**Table 1:** Mean CNR in the two groups with and without the MAR algorithm in different modes.

Mode	Group	Number	Mean	SD	*p* value
Ceramic brackets with coated archwire	MAR disabled	137	0.9697	0.6840	0.94
	MAR enabled	137	0.9789	0.6401	
	Total	274	0.9743	0.6612	
Ceramic brackets without coated archwire	MAR disabled	133	0.8472	0.6140	0.58
	MAR enabled	133	0.8025	0.5048	
	Total	266	0.8249	0.5618	

In the presence of ceramic brackets without coated NiTi archwire, the mean CNR is slightly lower when the MAR algorithm is enabled (0.8025 vs. 0.8472); however, the difference does not reach statistical significance (*p* = 0.58). In other words, activation of MAR has no significant effect on the artifact of CBCT images of teeth with ceramic brackets without coated archwire.

Table 2 presents the measures of central dispersion for the CNR of different teeth. As shown, a significant difference exists in this regard (*p* = 0.005), and the CNR in the region of the lateral incisor (1.0153) is the highest while the CNR in the region of the canine tooth (0.8272) is the lowest.

**Table 2:** Measures of central dispersion for the CNR of different teeth.

Tooth type	Number	Mean	SD	95% CI	
				Lower bound	Upper bound
Central incisor	108	0.9595	0.6368	0.8380	1.0810
Lateral incisor	102	1.0153	0.6860	0.8805	1.1500
Canine	104	0.7364	0.5272	0.6339	0.8389
First premolar	116	0.8272	0.5381	0.7282	0.9262
Second premolar	110	0.9696	0.6604	0.8448	1.0944
Total	540	0.9007	0.6183	0.8448	0.9530

As indicated in Table 3, a significant difference is found in CNR according to the distance from the tooth center (*p* = 0.009), such that the mean CNR increased by distancing from the tooth center. Mean CNR values in 15‐, 20, and 25‐mm distances are 0.8053, 0.8831, and 1.0033, respectively. Pairwise comparisons by the Bonferroni test (Table 4) shows that the mean CNR at 25 mm distance is significantly higher than that at 15 mm distance (*p* = 0.05).

**Table 3:** Mean CNR at different distances from the tooth center.

Distance (mm)	Number	Mean	SD	95% CI	
				Lower bound	Upper bound
15	168	0.8053	0.6069	0.7129	0.8977
20	184	0.8831	0.6012	0.7956	0.9705
25	188	1.0033	0.6326	0.9122	1.0943
Total	540	0.9007	0.6183	0.8484	0.9530

**Table 4:** Pairwise comparisons of different distances from the tooth center regarding CNR using the Bonferroni test.

			95% CI	
Distance (J)‐Distance (I)		Mean (I‐J)	Lower bound	Upper bound
15	20	0.0778	−0.2351	0.796
	25	−0.1980	−0.3545	−0.414
20	25	−0.1202	−0.2731	0.0327

## 4 Discussion

This study assessed the efficacy of a MAR algorithm for CBCT scans of teeth with ceramic brackets with/without coated NiTi archwires. The results showed that enabling the MAR algorithm had no significant effect on the CNR and could not decrease the metal artifacts. Thus, the null hypothesis of the study was accepted. The results also showed that distancing from the tooth center significantly increased the CNR, such that the CNR at 25 mm distance was significantly higher than that at 15 mm distance. Also, CNR was significantly different based on tooth type and was highest in the lateral incisor and lowest in the canine tooth.

CBCT is a crucial tool in orthodontics, providing detailed three‐dimensional images that aid in accurate diagnosis and treatment planning. However, the presence of metal artifacts, often caused by orthodontic appliances like brackets and wires, can significantly degrade image quality. These artifacts can obscure important anatomical structures, complicating measurements and diagnosis. For instance, in cases of impacted canines, clear visualization is essential for determining the precise position and planning the appropriate surgical or orthodontic intervention. MAR techniques, such as the use of specialized algorithms and software, are claimed to minimize these distortions and increase the image quality. However, the potential role of MAR algorithms for reducing or eliminating these artifacts has not been established yet. A search of the literature by the authors yielded no quantitative study on the efficacy of MAR algorithms for CBCT scans of teeth with ceramic brackets. However, the effectiveness of these algorithms for other diagnostic purposes has been previously investigated. Shavakhi et al. (2024) evaluated the effect of a MAR algorithm on CBCT images of teeth with stainless steel brackets and orthodontic archwires. They showed that MAR significantly increased the CNR and decreased artifacts only in the palatal position, and its effect was not significant on other positions. In line with their results, the present findings indicated that MAR is ineffective in increasing CNR in CBCT images in the presence of orthodontic brackets in the buccal position. It should be noted that they used Galileos Sirona CBCT scanner and its native MAR algorithm in their study while in the present study, the CBCT scanner and MAR algorithm of Papaya Genoray were employed. Oliveira et al. (2021) evaluated the diagnostic efficacy of a MAR algorithm for the detection of vertical root fractures and reported that it decreased the diagnostic accuracy. The difference between the findings of the study may be due to using a different CBCT scanner and algorithm in their work. They used a KaVo scanner while Papaya 3D was used in the current investigation. Moreover, the detection of vertical root fractures on CBCT scans is a challenging task. Additionally, Oliveira et al. used a qualitative index instead of CNR, which was applied in this study. Isman et al. (2020) evaluated the effect of orthodontic materials, FOV, and activation of MAR on the diagnostic accuracy of CBCT for the detection of proximal caries. They reported that MAR had no significant effect on diagnostic accuracy, similar to the present findings. This similarity may be due to using the same imaging protocol and the overall limited efficacy of the MAR algorithms in these circumstances. It should be mentioned that they used the Carestream CS 9300 CBCT scanner. Fontenele et al. (2020) evaluated the effect of a MAR algorithm on CBCT scans of a human mandible. They found that activation of MAR either before or after exposure significantly decreased artifacts, especially when the artifacts had a significant effect on image quality. Their result was in contrast to the present findings, which may be due to using different materials and differences in CBCT exposure settings. It should be noted that the software of Papaya 3D CBCT scanner used in the present study allows activation of MAR only after exposure.

In their study, Troca et al. (2024) have quantified the amount of artifacts from different orthodontic brackets and wires in CBCT images. Their results showed that all types of investigated orthodontic brackets (metallic, self‐ligating, and ceramic) create noise in the image. It should be noted that aluminum oxide brackets were used in the present study. Although due to the lower atomic number of aluminum than steel, these brackets cause less artifact than stainless steel brackets, assessment of the effect of their presence on CBCT image quality is important. Additionally, this study used only one FOV. The research conducted by Isman et al. (2020) demonstrated that the size of FOV does not influence the qualitative task of detection of caries in CBCT images, whether artifact reduction is applied or not. However, it remains unclear whether FOV would have impacted the quantitative analysis in this study, warranting further investigation using different FOV sizes.

In vitro design was the main limitation of this study, which limits the generalizability of the findings to the clinical setting. Future clinical studies are required to obtain more reliable results regarding the efficacy of MAR algorithms in the presence of ceramic brackets. Different CBCT scanners have unique exposure parameters and specifications, resulting in varying radiation doses and image characteristics. It is essential to point out that these variabilities may influence the amount of pre‐existing artifacts and the performance of metal artifact reduction models. Future studies should use other CBCT scanners and related software programs to assess the effect of activation of MAR before exposure. Furthermore, the efficacy of MAR and other deep‐learning‐based algorithms for reduction of artifacts caused by other types of brackets and archwires should be investigated.

The findings of this study contribute to the growing body of research on the challenges and limitations of CBCT imaging in the presence of metal artifacts. By identifying the effectiveness of MAR algorithms in particular diagnostic tasks and the specific factors that influence their effectiveness, this research can inform future development of image processing techniques and guide clinicians and radiologists in optimizing their use of CBCT for orthodontic diagnosis and treatment planning.

## 5 Conclusion

Within the limitations of this in vitro study, the results showed that the application of the MAR algorithm had no significant efficacy in improving the quality of CBCT scans of teeth with ceramic brackets with/without coated archwire.

## Author Contributions

Parisa Soltani supervised the study, participated in methodology, and wrote the initial draft. Mariangela Cernera participated in data analysis and wrote the initial draft. Marzie Kachuie designed the study, participated in methodology, and critically revised the original draft. Amirhossein Moaddabi helped in designing the study and wrote the initial draft. Mehran Khoramian participated in methodology and wrote the initial draft. Gianrico Spagnuolo interpreted the data and critically revised the original. Niccolò Giuseppe Armogida participated in data analysis and wrote the initial draft. Carlo Rengo participated in data analysis and critically revised the original draft. S.S. interpreted the data and critically revised the original draft. All authors approved the final manuscript.

## Ethics Statement

The study protocol was approved by the ethics committee of the Isfahan University of Medical Sciences (Number: IR.MUI.DHMT.REC.1403.044, Approval date: 10/6/2024).

## Consent

The authors have nothing to report.

## Conflicts of Interest

The authors declare no conflicts of interest.

## Data Availability

The data that support the findings of this study are available from the corresponding author upon reasonable request.
